# Mapping the distribution of *Amblyomma americanum* in Georgia, USA

**DOI:** 10.1186/s13071-024-06142-7

**Published:** 2024-02-11

**Authors:** Stephanie Bellman, Ellie Fausett, Leah Aeschleman, Audrey Long, Isabella Roeske, Josie Pilchik, Anne Piantadosi, Gonzalo Vazquez-Prokopec

**Affiliations:** 1 Gangarosa Department of Environmental Health, Rollins School of Public Health, Emory University, Atlanta, GA USA; 2 Department of Environmental Sciences, Emory University, Atlanta, GA USA; 3 Department of Pathology and Laboratory Medicine, School of Medicine, Emory University, Atlanta, GA USA

**Keywords:** *Amblyomma americanum*, Georgia USA, Tick, Species distribution, Predictive vector map

## Abstract

**Background:**

*Amblyomma americanum*, the lone star tick, is an aggressive questing species that harbors several pathogens dangerous to humans in the United States. The Southeast in particular has large numbers of this tick due to the combined suitable climate and habitats throughout the region. No studies have estimated the underlying distribution of the lone star tick across the state of Georgia, a state where it is the dominant species encountered.

**Methods:**

Ticks were collected by flagging 198 transects of 750 m^2^ at 43 state parks and wildlife management areas across the state from March to July of 2022. A suite of climate, landscape, and wildlife variables were assembled, and a logistic regression model was used to assess the association between these environmental factors and the presence of lone star ticks and to predict the distribution of these ticks across the state.

**Results:**

A total of 59/198 (30%) transects sampled contained adult or nymph *A. americanum*, with the majority of transects containing these ticks (54/59, 91.5%) in forested habitats. The presence of *A. americanum* was associated with elevation, normalized difference vegetation index (NDVI) on January 1, isothermality, temperature seasonality, and precipitation in the wettest quarter. Vast regions of central, eastern, and southern coastal Georgia (57% of the state) were categorized as suitable habitat for the lone star tick.

**Conclusions:**

This study describes the distribution of the lone star tick across the state of Georgia at a finer scale than the current county-level information available. It identifies specific variables associated with tick presence and provides a map that can be used to target areas for tick prevention messaging and awareness.

**Graphical Abstract:**

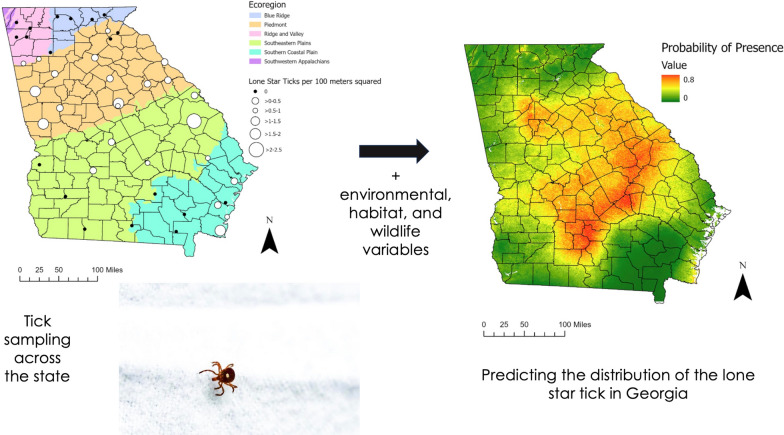

**Supplementary Information:**

The online version contains supplementary material available at 10.1186/s13071-024-06142-7.

## Background

Tick-borne disease (TBD) cases have been rising over the past decade in the United States, with approximately 20,000 reported in 2004 and more than 50,000 in 2019 [1, 2]. Although most of these cases are attributed to Lyme disease, other TBDs have also been on the rise, including spotted fever rickettsiosis, anaplasmosis, babesiosis, and ehrlichiosis. Additionally, there has been increasing incidence of alpha-gal syndrome, an emerging allergy to galactose-alpha-1,3-galactose present in red meat, associated with lone star (*Amblyomma americanum*) tick bites [3]. With this rise in emerging and established TBDs and pathologies, it is important to understand the areas at highest risk of human exposure to ticks in order to target communication and prevention efforts.

*Amblyomma americanum* has a wide geographical range in the USA, spanning the East Coast and Midwest [4]. This range has been expanding over the past few decades, with its native range in the Southeast expanding into the Upper Midwest/Northeast through multiple ecological mechanisms (e.g., climate change, host dispersal), and this expansion will likely continue in the future [5–7]. Lone star ticks are host generalists that feed on a wide variety of mammals, which also influences their ability to expand their range without specific host limitations [8]. These ticks parasitize humans at high rates; studies in the Southeast show that 53–83% of ticks found on humans are lone star ticks [9, 10]. Areas in the Northeast have also begun to see a rise in human contact with lone star ticks, with one study in New Jersey demonstrating *A. americanum* rapidly outpacing the previous most commonly encountered tick, *Ixodes scapularis*, over a period of 10 years [11].

While blood meal sources are important, ticks spend the majority of their life off-host and are therefore greatly affected by environmental factors [12]. One of the main risks to lone star ticks is desiccation, which influences the preferred habitats and regions suitable for the tick [13]. Researchers conducting ecological niche studies have used this knowledge to choose relevant climate and habitat variables to predict the suitability of different areas for *A. americanum* incorporating measures of temperature, precipitation, humidity, and landscape/land use [14, 15]. However, studies evaluating abiotic and biotic factors associated with the local distribution and abundance of *A. americanum* have had mixed results. Some researchers have found a lack of association between habitat and *A. americanum* [16, 17], while others have found strong links between forested habitat and *A. americanum* presence [18–20]. There are additionally no clear “best” climate variables for predicting *A. americanum* distribution, with studies finding significant relationships with a variety of different measures of temperature, precipitation, and humidity [14, 18, 21]. These differences may be influenced by regional heterogeneity in areas where the tick is being studied, highlighting the need to conduct more studies on smaller geographical scales. Identifying the specific factors influencing *A. americanum* distribution in an area is important for identifying locations most at risk for exposure to these ticks and the pathogens they carry. While most of this research in the USA has focused on the Lyme disease vector (*I. scapularis*) [22–24], *A. americanum* is an effective vector of lesser-known bacteria and viruses such as *Ehrlichia* species, *Rickettsia* species, Heartland virus, Bourbon virus, *Francisella tularensis*, and the agent of southern tick-associated rash illness [25, 26], and is not nearly as well studied [27]. These diseases are predicted to increase in burden as the lone star tick continues its northward range expansion and increases its opportunities for human biting [6, 11].

Though studies have been conducted to estimate the distribution of *A. americanum* in other states in the USA [18, 20], a comprehensive study assessing these factors has not been conducted in Georgia, a state in the native range of *A. americanum*, where it is the most commonly encountered tick [28, 29]. Work in Florida by Kessler et al. [20] demonstrated suitable habitat in the northern regions of the state near the Georgia border, finding associations with forest cover, isothermality, precipitation in the wettest month, mean temperature in the wettest quarter, precipitation seasonality, and maximum normalized difference vegetation index (NDVI). Given the increasing trends in the occurrence of alpha-gal syndrome in the state of Georgia [3], as well as the detection of Heartland virus in native *A. americanum* ticks in the state [30], developing detailed maps that quantify tick suitability has become a research priority. The objective of this study was to use climate, environmental, and habitat predictors collected at a wide array of natural and protected areas across all major Georgia ecoregions to model the distribution of *A. americanum*. Using this model, areas of Georgia can be identified as having a high likelihood for the presence of these ticks and targeted for sampling or intervention in the future.

## Methods

### Study site

This study was conducted across the entire state of Georgia. The state contains six level III ecoregions as designated by the Environmental Protection Agency (EPA), each characterized by unique vegetation, soil, climate, geology, and other biotic and abiotic factors creating distinct ecosystems [31]. Georgia has a humid subtropical climate, with elevation ranging from 1458 m in the Blue Ridge region to −3 m in the Southern Coastal Plain [32, 33]. It receives precipitation throughout the year, averaging 127 cm per year over the past century [34].

### Site selection

Sampling locations were chosen from Georgia state parks and wildlife management areas to target rural areas of human recreational exposure (e.g., hunting, hiking). To ensure coverage across the entire state, all state parks and wildlife management areas were assembled, and five sites per ecoregion were randomly selected (with the exception of the Southwestern Appalachians due to its small representation in Georgia). A further 18 sites were added to the initial 25 to fill geographical gaps and achieve broad coverage. Upon arrival, if sites were found to be inaccessible (flooded, road closures, etc.), the nearest comparable site was selected for sampling.

### Tick collections

Ticks were collected via flag sampling of 750 m^2^ transects measured using a measuring wheel and bounded with bright-colored stakes according to standard protocols [35]. Five transects, on average, were selected at each site, with each transect containing one habitat type. Replicates of habitat type (e.g., pine forest, deciduous forest, grassland) were determined based on the dominant vegetation at the site. For example, if a site consisted mostly of deciduous forest, multiple deciduous forest transects would be sampled. Transect locations were geotagged in Web Mercator (WGS84) in the center of the transect using an iPhone 13 (Apple Inc., Cupertino, CA, USA) and Google Maps (Google LLC, Mountain View, CA, USA). Transects were recorded with a unique identifier for the site, and transect numbers and pictures of the transect were taken for further habitat characterization. Collected ticks were placed in labeled plastic vials by transect and underwent microscopic identification of sex, species, and life stage using taxonomic keys [36, 37]. Collections took place between March and July of 2022, with the majority taking place between May and July to match the seasonality of *A. americanum* in Georgia [38, 39].

### Predictive variables

Predictors chosen for this study were based on previous *A. americanum* distribution models and spanned multiple categories including climate, landscape/land use, and wildlife ranges [14–16, 20, 40, 41]. For climate, all 19 bioclimatic variables were included from WorldClim version 2.1 at a resolution of approximately 1 km^2^ [42]. We additionally obtained meteorological data from the Goddard Earth Observing System Composition Forecasting (GEOS-CF) for Georgia from March to August 2022 [43]. The GEOS-CF data have a native resolution of 0.25°, and we further applied an inverse distance-weighted (IDW) interpolation using Euclidean distance to assign temperature and relative humidity values to each transect location on the day of sampling. Land cover was extracted using the 2019 National Land Cover Database (NLCD) at 30 m resolution via Google Earth Engine [44, 45]. Using field pictures and habitat characterization, a few transect points were reclassified when comparing the NLCD classification (e.g., open water to mixed forest when a point had been taken on the edge of a body of water). These classifications were additionally dichotomized into forest (deciduous, evergreen, and mixed) versus non-forest (all other categories) for variable selection. Elevation (NASA Shuttle Radar Topographic Mission [SRTM] v4, 90 m resolution) [46] and NDVI (MODIS [Moderate Resolution Imaging Spectroradiometer] V6.1, 16-day composites, 250 m resolution) [47] were extracted for each transect using the mean and median values, respectively, in a square buffer of 27 m (resulting in 729 m^2^ to mimic sampling methods) using Google Earth Engine. Lastly, utilizing the Georgia Department of Natural Resources Deer Harvest Dashboard, a deer density map by county was included in our variable selection [48].

### Statistical methods/modeling

The dichotomized presence or absence of *A. americanum* adults and nymphs in each transect was used as the outcome variable for distribution modeling. Both life stages were counted together because both were found in the majority of transects, and we were not interested in a particular life stage. The transects were then split at random into training (85% of transects) and testing (15%) datasets for model evaluation. This split was chosen due to the small overall number of transects, to maximize transects used for model development. The locations of the training and testing datasets were representative of each ecoregion in Georgia. For training, 19 transects were in Blue Ridge, 20 in Ridge and Valley, 62 in Piedmont, 43 in the Southeastern Plains, and 29 in the Southern Coastal Plain. Of the total testing locations, the relative representation of each ecoregion was 14% (3/22) for Blue Ridge, 20% (5/25) for Ridge and Valley, 5% (3/65) for Piedmont, 14% (7/50) for the Southeastern Plains, and 19% (7/36) for the Southern Coastal Plain.

All potential predictors were included in the model, and backward selection was conducted using the stepAIC function in the MASS package (v7.3-54) in R (version 4.0.4). Interaction terms involving forested habitats were also assessed. Once an initial set of predictors were dropped through this function (minimizing the Akaike information criterion [AIC]), collinearity was assessed using the CAR package (v3.0-12), and additional predictors were dropped by collinearity size (variance inflation factor [VIF] > 10) and least statistical significance. The final model followed the above criteria, stopping when all predictors had VIFs < 10 and when AIC no longer decreased upon variable removal. The selected final model was then assessed for predictive accuracy and fit using the testing dataset. Continuous predicted values for the probability of *A. americanum* presence were generated using the final model on the testing set. To dichotomize this predicted probability, the optimal cut point was determined using the OptimalCutpoints package (v1.1-5), maximizing the efficiency of the model. Additionally, a receiver operator characteristic (ROC) curve was generated to compare the optimal cut point computed with the sensitivity and specificity relationships on the curve. Values above the cut point were classified as presence, while those less than or equal to the cut point were considered absence. The sensitivity, specificity, accuracy, area under the ROC curve (AUC), and kappa values were calculated for the model on both the training and testing datasets.

### Mapping

Raster surfaces of all predictors and point locations of all transects were imported into ArcGIS Pro (v2.8.0). Collected tick totals were represented as density per 100 m^2^ sampled according to the Centers for Disease Control and Prevention (CDC) metastriate tick surveillance guide recommendations [35]. The density of *A. americanum* per 100 m^2^ sampled at each site was calculated by taking the total *A. americanum* adults and nymphs collected at the site, dividing by the total distance transected at the site, and multiplying by 100. For the predictive maps, the Raster Calculator function in the Spatial Analyst extension was used to enter the model statement using the coefficients generated in R and the raster surfaces corresponding to the variables in R. The Raster Calculator function was used again with the formula 1/(1 + Exp[−1×model]) to generate a continuous probability surface at a spatial resolution of approximately 1 km^2^ for the niche of the tick in Georgia based on the model. This probability map output was additionally dichotomized in another map using the ROC cutoff value to indicate areas that would be categorized as suitable habitat for *A. americanum* ticks.

## Results

**Fig. 1:**
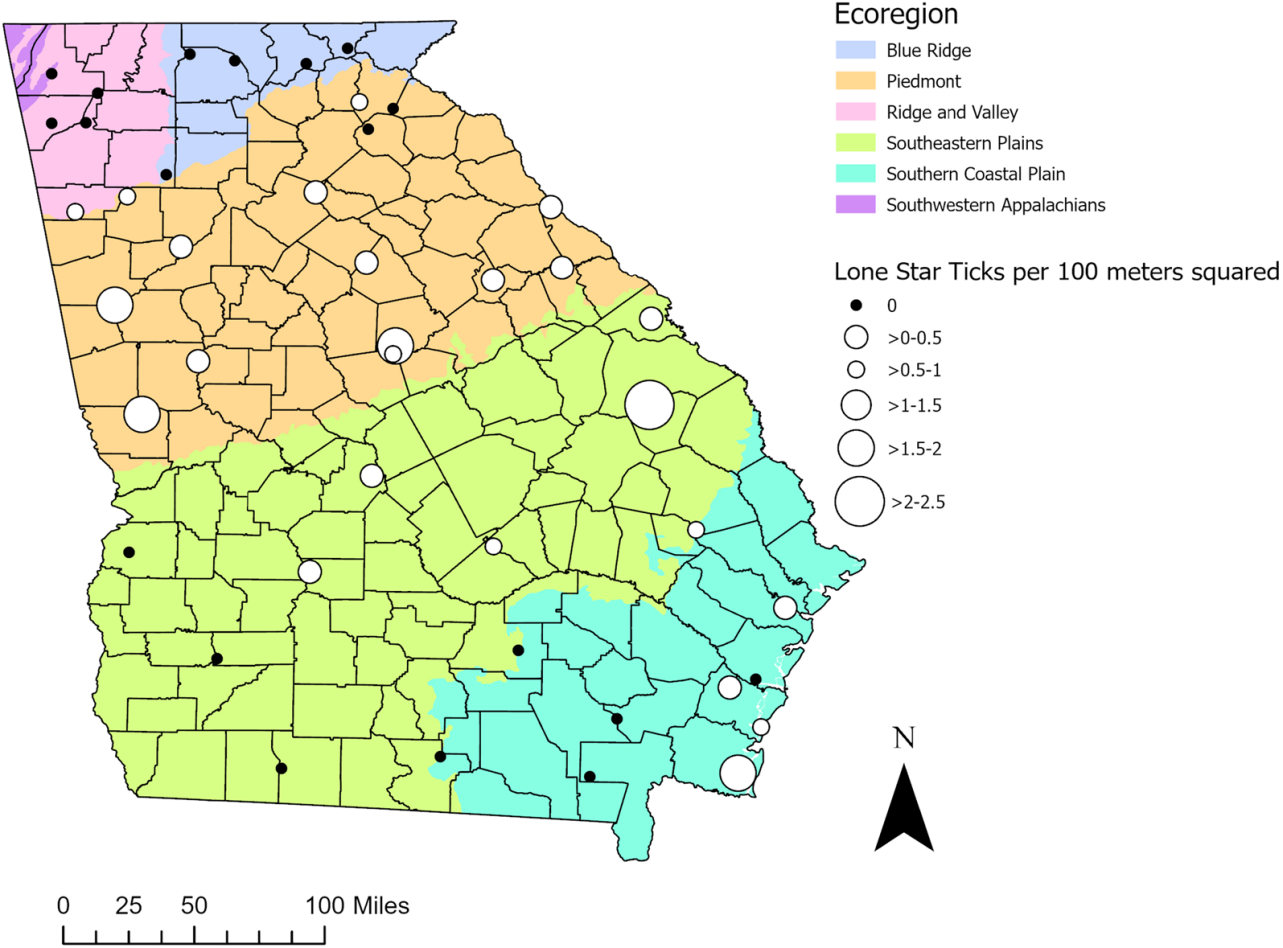
Density of *A. americanum* at each site per 100 m^2^ sampled. The total number of *A. americanum* collected at each site was divided by the total transected distance and multiplied by 100 to display the density of adult and nymph *A. americanum*. Larger white circles represent sites with higher density of lone star ticks

In total, 630 ticks were collected in 198 transects at 43 locations in Georgia. Of these, 568 (90%) were either *A. americanum* adults or nymphs, 24 were *I. scapularis* adults, 30 were *Dermacentor variabilis* adults, and eight were *Amblyomma maculatum* adults. Fifty-nine of the 198 transects sampled had adult/nymph *A. americanum* present, with totals ranging from 1 to 77. *Amblyomma americanum* density per site ranged from 0 to 2.43 ticks per 100 m^2^ sampled (Fig. 1).

**Fig. 2:**
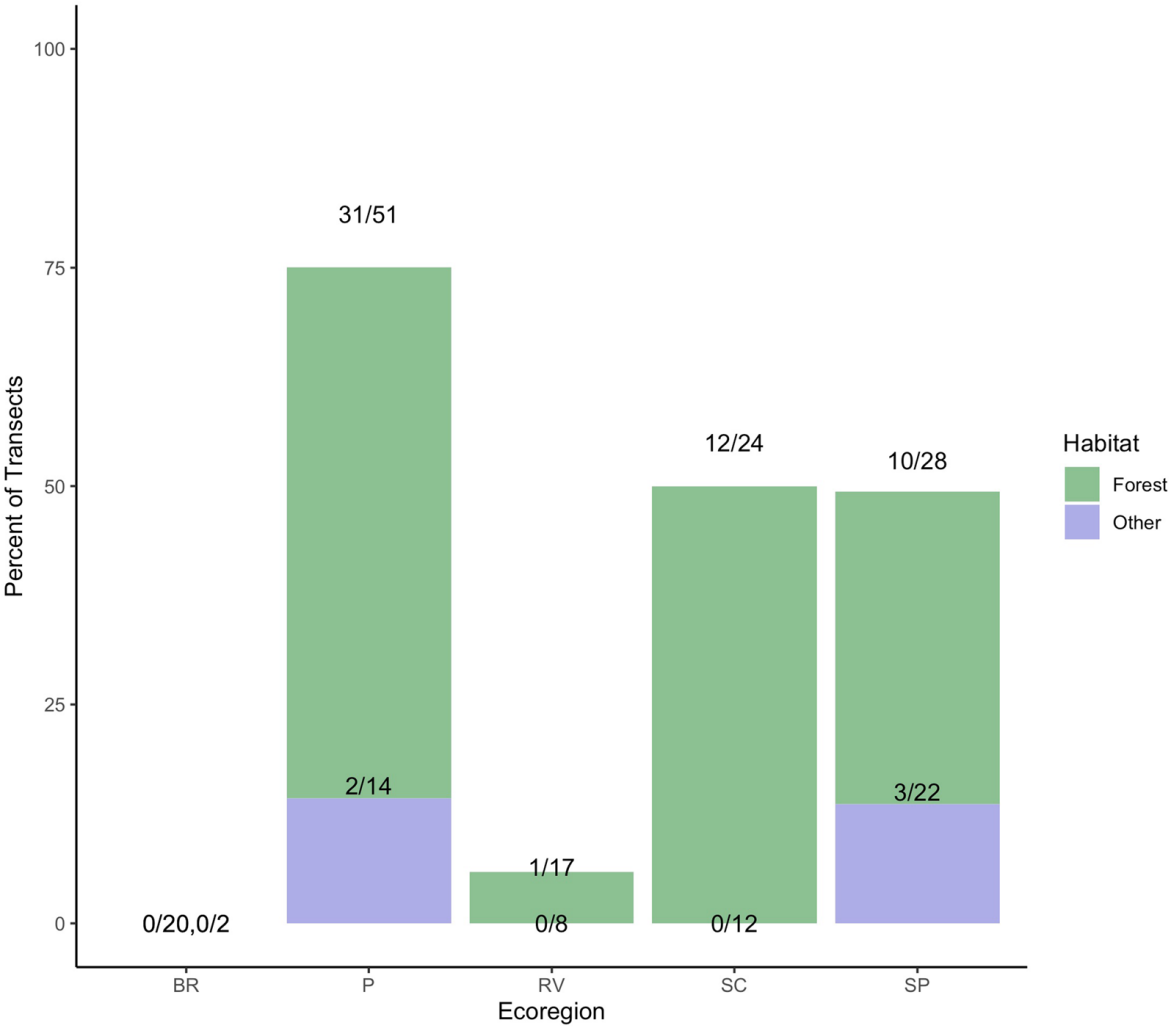
Bar graph of the percentage of transects where *A. americanum* was found, split by habitat in each ecoregion (*BR* Blue Ridge, *RV* Ridge and Valley, *P* Piedmont, *SP* Southeastern Plains, *SC* Southern Coastal Plain). In total, 0/20 (0%) forested transects contained lone star ticks in Blue Ridge, 31/51 (60.8%) in Piedmont, 1/17 (5.9%) in Ridge and Valley, 12/24 (50%) in the Southern Coastal Plain, and 10/28 (35.7%) in the Southeastern Plains. For grassland/other transects, 0/2 (0%) had lone star ticks in Blue Ridge, 2/14 (14.3%) in Piedmont, 0/8 (0%) in Ridge and Valley, 0/12 (0%) in the Southern Coastal Plain, and 3/22 (13.6%) in the Southeastern Plains

The majority of transects sampled (140/198, 70.7%) were categorized as forest in the field (deciduous, mixed, or pine), with percentages of forest versus other transects ranging from 56% of transects in the Southeastern Plains ecoregion to 91% of transects in the Blue Ridge ecoregion. In ecoregions where *A. americanum* ticks were found (all but the Blue Ridge region), the majority were found in forested transects (Fig. 2). More than one third—54 of 140 (38.6%)—of forested transects sampled contained *A. americanum*, while only five of 48 (10.4%) grassland transects contained *A. americanum*. In other words, in transects where *A. americanum* ticks were found (*n* = 59), 91.5% of these were forested (*n* = 54). Clustering of *A. americanum* was also more common in forest habitats, with around 10 ticks per tick-containing transect, versus around three ticks per tick-containing transect in grassland habitats. Although forest appears to be descriptively significant, this effect was not seen during the modeling phase, likely due to the differences in *A. americanum* presence in forests across the ecoregions of the state (Fig. 2).

### Model characteristics

After initial backward selection, 13 bioclimatic variables, temperature on the day of sampling, the NDVI value on January 1, and elevation were included in the final model selection. The final model deleted predictors to reduce VIF to < 10 while also minimizing AIC increases (Additional file 2: Table S1). Predictors present in the final model were elevation, NDVI on January 1, Bioclim 3 (isothermality), Bioclim 4 (temperature seasonality), and Bioclim 16 (precipitation in the wettest quarter) (Table 1). All three bioclimate variables were negatively associated with tick presence. Thus, as isothermality, temperature seasonality, and the amount of precipitation in the wettest quarter increased, the likelihood of finding *A. americanum* decreased. Given the more difficult interpretation of isothermality, we show in Additional file 2: Fig. S1 the relationship between Bioclim 3 and the probability of detecting *A. americanum*, whereas Additional file 2: Fig. S2 shows the distribution of isothermality across Georgia. Elevation and the NDVI on January 1 were positively associated with tick presence, so as the elevation or NDVI value increased, the likelihood of finding *A. americanum* ticks increased (Table 1).

**Table 1:** Model predictors and coefficients for the best logistic regression model

	Beta	Std error	*Z*-value	Pr ( >|z|)	Significance
Intercept	34.792	11.369	3.060	0.002	**
Bioclim 3	−0.287	0.115	−2.506	0.012	*
Bioclim 4	−0.018	0.008	−2.266	0.023	*
Bioclim 16	−0.032	0.008	−3.965	7.35e−05	***
Elevation	0.003	0.002	1.448	0.148	NS
NDVI (Jan 1)	0.0002	0.0002	0.917	0.359	NS

***P <0.0001, **P <0.001, *P <0.01, *NS* not significant

The model performed better on the testing dataset than on the training dataset (Table 2). Although the model had good sensitivity on the training set (ability to predict areas of tick occurrence that truly had ticks when sampling), this decreased when applied to the testing dataset. The reverse was true for specificity; the model performed better at calling areas that did not have *A. americanum* ticks (when they did not have ticks upon sampling) for the testing dataset than the training dataset. Two transects in the training set had missing values for NDVI on January 1 and were dropped from predictive calculations in the model (FMT3 and JET5, both located in the Southern Coastal Plain ecoregion). The kappa value is an accuracy statistic that takes into account random chance. The value for the training set (0.33) indicates that the model performance is “fair,” while the level of agreement for the testing set is “moderate” [49]. The AUC was also computed, yielding 70% for the training set and 80% for the testing set.

**Table 2:** Model performance

	Accuracy	Sensitivity	Specificity	Kappa	AUC
Training (*n* = 171)	0.66	0.80	0.60	0.33	0.70
Testing (*n* = 25)	0.84	0.71	0.89	0.60	0.80

### Distribution maps

**Fig. 3:**
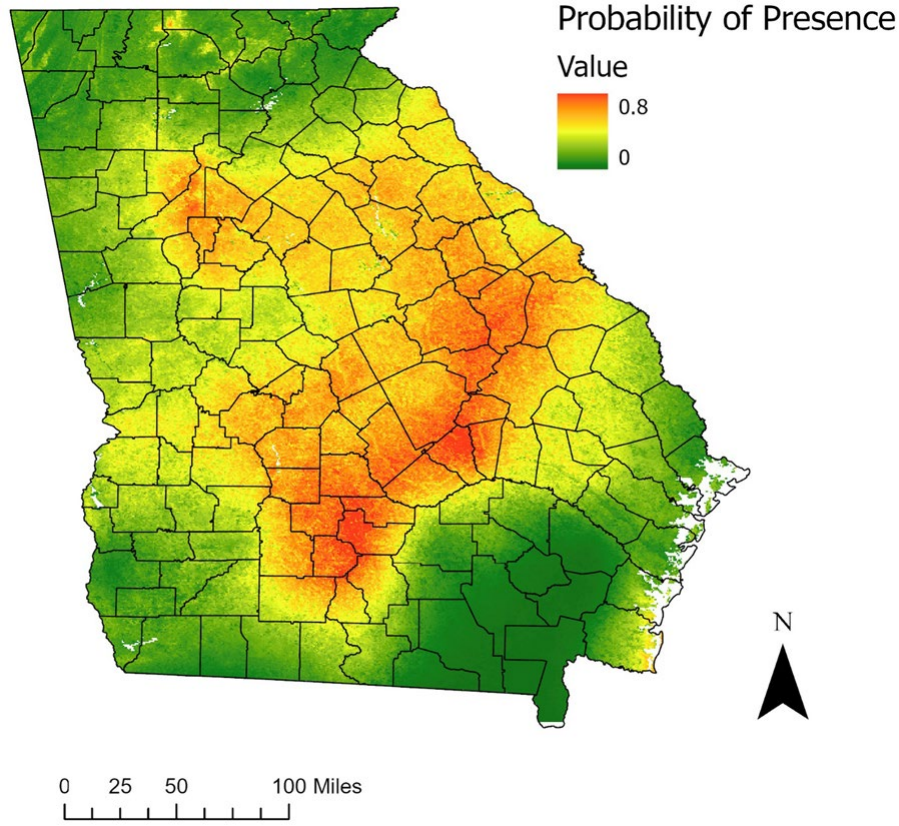
Probability of *A. americanum* occurrence across Georgia, estimated from the best-fit logistic regression model of tick presence. Areas that are yellow to red indicate areas with a higher probability of finding questing lone star ticks. White reflects missing data

The map of the probability of *A. americanum* presence demonstrates that the areas at highest risk of encountering this tick are in eastern and central Georgia and the southern edge of the coast (Fig. 3). There were missing data for the rest of the coast. Areas less suitable for questing *A. americanum* included large parts of northern and southeastern Georgia. Because of the missing data for some of the coastal areas, no prediction was made for some of this region, though where present on the southern edge of the coast, increased suitability was projected.

**Fig. 4:**
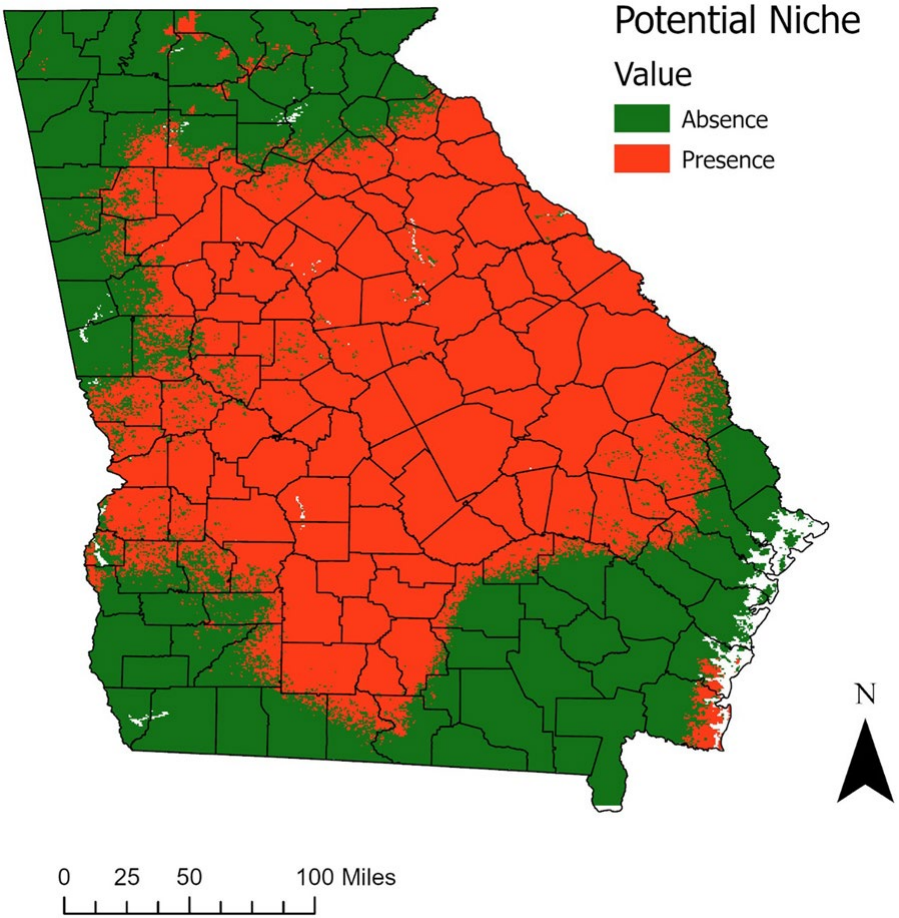
Estimated ecological niche of *A. americanum* in Georgia using the optimal probability cut point maximizing sensitivity and specificity (0.2508). Red areas indicate locations that would have suitable habitat for *A. americanum* based on the final model

The map was further categorized into presence/absence of the tick based on the most efficient cut-off point in the model (0.2508), and this map reveals all areas where *A. americanum* can be expected to be present and questing (Fig. 4). The areas indicated as suitable habitat span large swaths of the Piedmont and Southeastern Plain ecoregions, with some additional locations along the Southern Coastal Plain and the edge between the Ridge and Valley and the Blue Ridge ecoregions [31]. In total, 57% of the area of Georgia was predicted to be suitable for *A. americanum* questing.

## Discussion

TBDs constitute the majority of vector-borne illnesses reported in the United States and have been on the rise over the past century [2]. Though many studies have been devoted to characterizing the distribution of *I. scapularis* and Lyme disease in the Northeast [23, 50–52], there has been less focus on other tick species such as *A. americanum* and its pathogens in the Southeast, where it is the dominant tick species [53]. Understanding the distribution of this medically important vector is crucial to identifying areas at highest risk of tick contact for targeting of TBD prevention resources and messaging.

In this study, we conducted intensive field sampling across the state of Georgia to identify locations with questing *A. americanum*. Incorporating remote sensing data and other biologically important variables, we created a logistic regression model to predict areas with the highest suitability for nymph and adult life stages of *A. americanum*. The model identified large regions of central, eastern, and southern coastal Georgia as having the highest probability of containing lone star ticks. Before this study, the only information on *A. americanum* species distribution in Georgia was aggregated at the county level, and many counties, especially in the southern half of the state, were missing data [28, 41]. Georgia presented one of the largest data gaps in the Southeast [41], and our study and maps show that using regional tick presence data overestimated the suitability of Georgia for *A. americanum*. The distribution maps created here can be used to estimate areas where people may encounter questing lone star ticks in Georgia. They can guide epidemiological studies quantifying the role of *A. americanum* in human illnesses.

Consistent with other literature, the majority of *A. americanum* collected in this study were found in forested habitats [18–20]. Although the tick is considered to be a habitat generalist [17] and is found in some grassland transects in this study, most transects with *A. americanum* were deciduous, pine, or mixed forests. Interestingly, however, being a forested habitat was not a predictor included in the final model and was not statistically significant in model selection. This is likely because forest habitat alone does not guarantee that *A. americanum* ticks will be present, and other factors such as climate, elevation, and wildlife density contribute more strongly to habitat suitability. In our sampling, we primarily surveyed forested transects (140 of 198) in Georgia, but in the two northern ecoregions (Blue Ridge and Ridge and Valley), *A. americanum* was present in only 3% of these forested transects, while in the other three regions (Piedmont, Southern Coastal Plain, Southeastern Plains), it was found in 51% of forested transects (Fig. 2). Although the interaction terms tested using habitat and ecoregion were additionally nonsignificant, this example, consistent with other studies [16, 54], demonstrates that factors other than general habitat are crucial in determining the *A. americanum* niche.

The predictors present in the final selected model were elevation, NDVI on January 1, Bioclim 3 (isothermality), Bioclim 4 (temperature seasonality), and Bioclim 16 (precipitation of the wettest quarter). A comparable distribution modeling study was conducted previously by Kessler et al. [20] in Florida, and their model contained a related suite of final variables including forest cover, isothermality, precipitation in the wettest month, mean temperature in the wettest quarter, precipitation seasonality, and maximum NDVI. Florida shares two of Georgia’s six level III ecoregions, the Southeastern Plains and Southern Coastal Plain [31], and shares similar weather patterns to Georgia in the northern parts of the state. Though largely similar, Georgia has a wider range of climate and elevation through its six ecoregions, which likely underlies some differences in predictors and model performance between the studies.

The variables included in the final model of this study can be related to important characteristics with regard to *A. americanum* survival. Both isothermality and temperature seasonality relate to variability in temperature, the first characterizing the daily temperature range compared with annual temperature range, while the second is based on the standard deviation of monthly temperature averages [42]. Higher isothermality values indicate smaller annual seasonal temperature changes relative to diurnal temperature fluctuations and can be found in southeastern Georgia, while lower isothermality values are indicative of larger differences between summer and winter temperatures than daily temperatures and can be found in central, coastal, and northern Georgia (Additional file 2: Figs. S1 and S2). For temperature seasonality, the higher the value, the more variability there is in monthly temperature averages over the year (seen in areas of central and northern Georgia), and the lower the value, the less variability in temperature across the year (seen broadly across southern Georgia). These seasonality factors have been indicated as important predictors in other *A. americanum* distribution studies [20, 55], and temperature range is important to tick survival off-host [56]. In our model, both isothermality and temperature seasonality were negatively associated with tick presence when accounting for the other variables, indicating that the probability of finding *A. americanum* ticks decreased with increased seasonal temperature variability (temperature seasonality) and larger diurnal temperature fluctuation compared with annual temperature range (isothermality). Lone star ticks are sensitive to freezing and desiccation seen with temperature extremes [57, 58], so both high fluctuations in diurnal temperatures and high variability in annual temperatures may create suboptimal conditions and reduce habitat suitability.

Precipitation is also important to *A. americanum* tick survival, as humidity has been shown to be an important factor in the *A. americanum* life cycle [57, 59]. Precipitation in the wettest quarter was negatively associated with the probability of tick presence, indicating that the more precipitation an area received in the wettest three months of the year, the less chance lone star ticks were present. Georgia receives ample precipitation year-round [34], so areas with the highest precipitation are likely more prone to flash flooding and swamp-like habitats (favored by high amounts of clay in the soil), potentially impacting oviposition and egg hatchability in early tick life stages [60] and decreasing questing behavior [61].

Elevation was positively associated with the probability of *A. americanum* presence when accounting for other variables in the model. This association may be counterintuitive, as *A. americanum* is more commonly found at lower elevations [62], and it was not found in our field collections in the highest-elevation ecoregion in Georgia (Blue Ridge). However, this association is likely due to the inclusion of the other variables in the model. The univariate relationship between elevation and tick presence was negative but changed to positive when adding the climate and NDVI variables to the model. The climate variables had stronger associations with tick presence than elevation in this dataset and, because of this, may better explain outside characteristics associated with elevation (e.g., weather patterns in mountainous areas) that impact tick habitat suitability. Therefore, elevation, when accounting for NDVI on January 1, isothermality, temperature seasonality, and precipitation of the wettest quarter, shifts to a slight positive association to describe smaller regional nuances in the data.

The NDVI value measured on January 1, 2022, was positively associated with tick presence in the model. NDVI is a measure of vegetation health, with higher values indicating healthy vegetation [63, 64]. These values are lower in winter months than in summer months, as many plants undergo senescence in the colder months, losing leaves, flowers, fruits, etc. [65]. Areas that have higher NDVI relative to others during the winter are likely locations with some evergreen composition to their forest, as they would be less affected during these months [66]. Therefore, pine and mixed forests (higher NDVI values in January) were associated with higher probability of *A. americanum* presence. Pine forest microclimates are generally hotter and drier than deciduous forest microclimates, and as a result, ticks questing in the summer in these habitats experience more desiccating conditions [54]. As previously mentioned, *A. americanum* have not been exclusively associated with a single habitat in the literature [17] and have the ability to quest in drier conditions due to the presence of a waxy cuticle [67]. Although we did not find a significant association between forested habitat and tick presence, NDVI in January may represent similar factors relating to the health of vegetation and pine/mixed forest habitats.

Modeling studies on habitat suitability and vector distribution are only as good as the data underlying the model and the types of data available for predictive selection [68]. In this study, although we traveled to 43 locations in the state, there were still sections of Georgia that could not be covered due to size and limitation by locations of state parks and wildlife management areas. Additionally, there may be practices in these parks that impact tick populations, such as controlled burns [69], that we were not aware of during sampling and were unable to account for in our model. Furthermore, ticks are not equally distributed in the environment and are frequently clustered, which may, by chance, not have been selected within a given transect [70]. To combat this, multiple transects were conducted at each site and free flagging was conducted outside of transects to validate our results. Despite this, there is still a possibility that ticks were present in areas we sampled but we failed to detect them in our flagging. There may also be variables we are unable to include in our model that are important to tick abundance, such as wildlife movement and density, which would impact host availability for ticks.

The maps created in this study can be leveraged for additional lone star tick sampling for active surveillance across the state and for targeting areas for tick protection messaging. To improve upon the initial work done here, future distribution studies could utilize additional modeling techniques (machine learning techniques such as random forest models or MaxEnt [maximum entropy]) to create a composite result [21, 71]. Further work could characterize the other tick species distributions in Georgia by sampling during their peak seasons (e.g., *Ixodes* in spring and fall) to more completely describe the tick landscape in the state. Lastly, this map may be compared with TBD cases in the state to assess associations between counties at highest risk of the presence of lone star ticks and county rates of different associated diseases and pathologies (e.g., ehrlichiosis, alpha-gal) to further understand how areas with high tick suitability translate to human disease cases [18].

## Conclusions

This study generated a predictive map of the State of Georgia to identify areas with highest habitat suitability for *A. americanum*. This map can be used to estimate where people are at highest risk of encountering questing *A. americanum* and target these areas for tick prevention messaging.

### Supplementary Information

**Additional file 1: Dataset S1.** *Amblyomma americanum* outcome data and predictors used for modeling.

**Additional file 2. **Additional file tables and figures.

## Data Availability

The datasets used and analyzed during the current study are available in Additional file 1: Dataset S1 included in this publication.

## Abbreviations

NDVI: Normalized difference vegetation index
TBD: Tick-borne disease
GEOS-CF: Goddard Earth Observing System Composition Forecasting
NLCD: National Land Cover Database
AIC: Akaike information criterion
VIF: Variance inflation factor
ROC: Receiver operator characteristic
AUC: Area under the curve
