# Supplementary material for: Mapping the distribution of Amblyomma americanum in Georgia, USA
Source: Parasit Vectors. 2024 Feb 11;17:62. doi: 10.1186/s13071-024-06142-7 (PMC10860309; doi:10.1186/s13071-024-06142-7)
Supplement: Supplementary file 2 — Additional file 2. Additional file tables and figures. [file 13071_2024_6142_MOESM2_ESM.docx]

Mapping the distribution of *Amblyomma americanum* in Georgia, USA

Additional file Tables and Figures

Table S1. Table of collinearity (VIF) in the training set (after initial backward selection but before dropping variables due to collinearity)

| Variable | Variance Inflation Factor (VIF) |
| --- | --- |
| Bc1 | 11186.68 |
| Bc2 | 536.32 |
| Bc3 | 299.96 |
| Bc4 | 7332.95 |
| Bc5 | 261.50 |
| Bc9 | 2.96 |
| Bc10 | 8352.62 |
| Bc11 | 24674.82 |
| Bc13 | 181.44 |
| Bc14 | 73.42 |
| Bc16 | 263.58 |
| Bc17 | 89.44 |
| Bc19 | 30.54 |
| elevation | 81.11 |
| J1NDVI | 1.58 |
| t | 2.50 |

Abbreviations: bc# - Bioclim # variable from WorldClim, J1NDVI – NDVI value on January 1, 2022, t – temperature on date of sampling at transect location

Figure S1. Predicted effect of bioclimatic variable isothermality (bioclim3/bc3) on the presence of *A. americanum* in Georgia (ama_bin), after accounting for additional factors in the model. The band represents the 95% CI of the linear estimate (line). Presence is expressed as probability of detection.

**
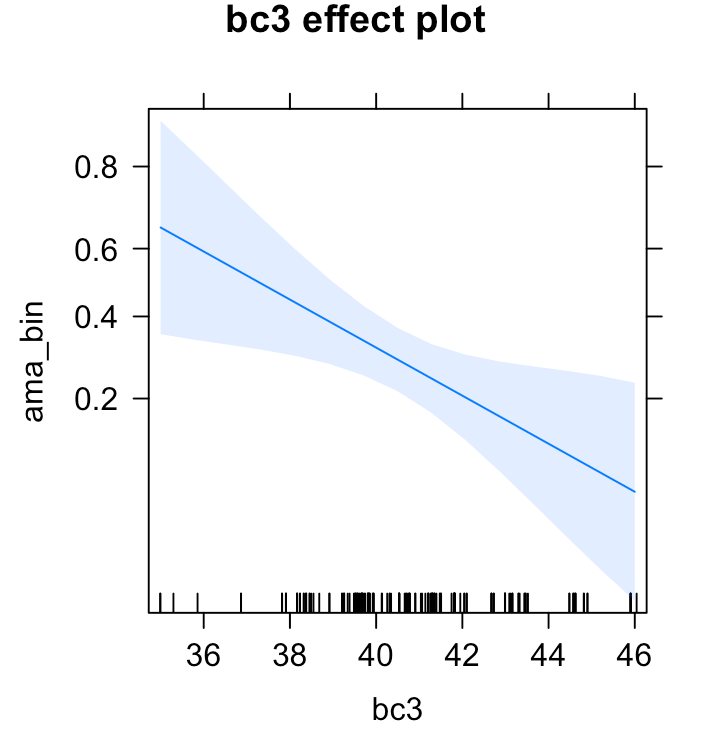
**

Figure S2. Map of isothermality (bc3/bioclim3) across Georgia. Darker colors indicate higher values for isothermality.

**
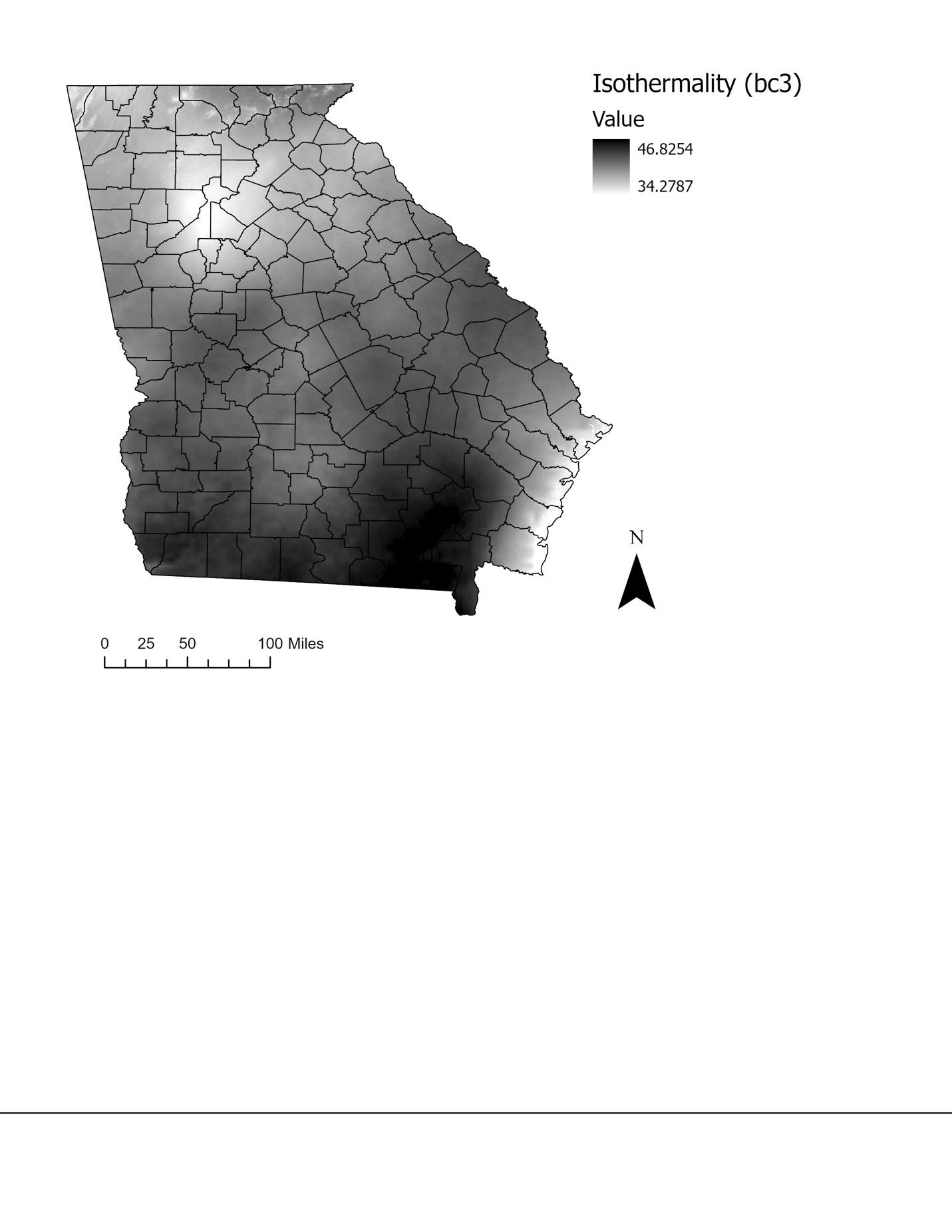
**
